# Is Every Law for Everyone? Assessing Access to National Legislation through Official Legal Databases around the World

**DOI:** 10.1093/ojls/gqac032

**Published:** 2023-02-04

**Authors:** Andreas Nishikawa-Pacher, Hanjo Hamann

**Affiliations:** Recipient of a DOC Fellowship of the Austrian Academy of Sciences at the Department of Legal and Constitutional History, University of Vienna, Austria; Vienna School of International Studies, Austria; EBS University of Business and Law, Wiesbaden; Institute for Globally Distributed Open Research and Education

**Keywords:** empirical legal studies, access to law, legal databases, law and technology, rule of law

## Abstract

Countries all over the world document their statutory law in official legal databases (OLD), but the extent to which these provide effective access to (statutory) law remains unexamined. Ideally, an OLD should be (i) provided online and free for all without requiring registration or payment, (ii) searchable with regard to statutes’ titles, (iii) searchable with regard to the full texts of statutes, (iv) provided in a reusable text-based format and (v) comprehensive in its coverage of at least the laws currently in force. To highlight the nature of OLDs as consumer products, we borrow a term from business operations research and refer to a database fulfilling these basic criteria as a ‘minimum viable’ OLD. We survey 204 states and jurisdictions to assess how far their country-level OLDs adhere to the minimum viability standard. We find that only 48% of them do; 12% of states do not seem to offer any online OLD at all; and a further 40% of countries offer legal databases that lack at least one of the criteria listed above. The quality of legal access is associated with geographical distribution (with Europe faring the best), economic development and a population’s overall Internet usage. The results suggest that comparative legal research faces considerable hurdles when dealing with the Global South; that metadata-enriched digitalisation of legal corpora still remains a desideratum for at least half the world; and that the inaccessibility of law may carry high costs for legal practitioners and the wider public.

## 1. Introduction

Human rights scholars are increasingly acknowledging a new fundamental right in this digital era: the ‘right of public access to legal information’ or, more narrowly, the ‘right of access to law’.^1^ Such access to law, it may seem, could easily be achieved by documenting statutes or case law online. Yet, pioneers of open legal data have long noted that there is more to making law *accessible* than merely getting it *online*.^2^ While some governments grant easy access to their sources of law through online portals with advanced search options, the extent to which this is common has never been studied systematically on a global scale. Thus, it remains unknown whether effective infrastructures are a ubiquitous reality or a rare exception. Even if they turn out to be widespread, there may be great variability in the quality of legal databases.^3^ Some countries may offer free and comprehensive access to legal texts even in machine-readable formats, others may offer only a rudimentary list of non-searchable, low-quality image files, while yet other countries may not provide any systematic access to legal texts at all. We encountered all of these prototypes in our research, but the extent of such variation and its implications to access to law have never been studied systematically.

Why should legal scholars care about an apparent technicality, namely about how legal sources—specifically statutes, as the focus of the present study—get provided to the public? In the remainder of this introduction, we will first answer this normative question, then explain the data collection effort which we document in subsequent sections.

### A. The Case for Effective Access to Statutory Law

There are at least three prongs to a normative argument about the accessibility of statutory law. The simplest normative argument derives from the ancient maxim *ignorantia legis neminem excusat* (‘none shall be excused by their ignorance of law’), which is still a fundamental principle of justice in most legal systems. While the principle appears somewhat paradoxical in that we cannot expect every subject of law to have read and memorised each legal rule, there are neurobiological and psychological explanations of how laws pervade their respective communities and get sufficiently internalised to secure general law-abidingness.^4^ All of these explanations necessarily start with some multiplier’s first-hand knowledge of the law, and a medium to transmit it to other members of society. This means that in an information society, there is just one practical way of complying with the normative principle: ‘if ignorance of the law is no excuse, then there is an obligation to publish the law’,^5^ and in a manner enabling effective dissemination. Similar arguments can be traced to prominent proponents of the rule of law (such as Raz and Fuller) and have been more fully developed in studies of the ‘social dimension’ of the rule of law.^6^

Another argument derives from public finance considerations, namely the fact that in most legal systems statutes are drafted, discussed and decreed based on taxpayers’ money, without which the entire bureaucratic machinery limiting individuals’ freedoms for the sake of public interests could not operate. Where subjects of the law fund the state forging their shackles, the argument goes, subjects of the law are entitled to effective means of ascertaining their state’s laws. As a poignant catchphrase, this might be shortened to: ‘Law Belongs to the People’.^7^ Similarly, the work of legal information institutes is based on a Declaration on Free Access to Law, which recognises legal information as ‘part of the common heritage of humanity’ and ‘digital common property’.^8^

The third normative argument is instrumental in nature and considers access to law as a ‘means to an end’ for the specific functions of law, arguing that both its ‘control and regulation aspect’ and its ‘facilitating aspect’ imply the existence of a ‘cognisable legal basis’.^9^ While we cannot reiterate the entire argument here (and need not, since it has been expounded eloquently by others),^10^ we note that its underlying premise has been endorsed, *inter alia*, by justices at the European Court of Human Rights, who have argued for decades that

the law must be adequately accessible: the citizen must be able to … regulate his conduct: he must be able—if need be with appropriate advice—to foresee, to a degree that is reasonable in the circumstances, the consequences which a given action may entail.^11^

### B. The Current State of Effective Access to Statutory Law

Existing assessments of the online accessibility of e-government suggest that access to statutory law might be scarce in many parts of the world. Most such studies, however, narrow their focus to the *judicial* branch while neglecting *legislative* powers.^12^ When it comes to legal statutes, it seems that only the Global Open Data Index (GODI)^13^ has attempted an international survey, although no scholarly publications seem to have resulted from this exercise. Considering 94 countries, GODI measured the availability of legal statutes based on six open data principles, namely whether domestic laws are: (i) openly licensed; (ii) formatted in a machine-readable way; (iii) downloadable at once; (iv) up to date; (v) publicly available; and (vi) free of charge. GODI’s most recent survey, from 2016, found that only 10 countries satisfied all six principles, while the majority of jurisdictions surveyed were rated with less than half the total score. The other half of the world (outside the 94 jurisdictions surveyed) was disregarded because they had not reached GODI’s threshold of participation: they had failed to provide online information about the content of the law, about the respective date of last amendment and about any amendments to each law. GODI’s results and limited sample size thus already attest to a potentially bleak state of legal access, albeit at a point in time several years ago.

Some private initiatives have thus sprung up to fill the gap in providing access to law. The Constitute Project^14^ provides a notable example: it offers a structured, metadata-enriched corpus of 201 contemporary and historical constitutions with English translations. However, its utility for evaluating a broader, comprehensive access to binding statutes remains limited, as it confines itself to constitutions. When it comes to legal acts below the constitutional level, one can find private initiatives that deal with single countries. For instance, the Open Knowledge Foundation (OKFN) sponsors a project in Germany that regularly re-releases all contents of the Federal Law Gazette (*Bundesgesetzblatt*) on a public website that follows open data principles.^15^ There are also platforms that collect laws from more than one country, with the most ambitious being the university-based project World Legal Information Institute (WorldLII)—its ultimate goal is to maximise the availability of public legal information from every country on a single platform for anyone to use for free.^16^ While such initiatives do fill crucial gaps, they do not offer suitable substitutes for official gazettes and governmental databases.^17^ They will always lack official authority; they cannot rule out incomplete coverage;^18^ they cannot guarantee to be timely; and their funding is less secure and sustainable than if they belonged to a state agency.^19^ In their ‘Disclaimers of Liability’, WorldLII rightly states that it

does not give any guarantees … concerning the accuracy, completeness or up-to-date nature of the information provided on WorldLII. Users should confirm information from another source if it is of sufficient importance for them to do so. For official versions of legal reference materials, users should refer to their respective official publications.^20^

In contrast, commercial platforms (such as LexisNexis or WestLaw) may provide more reliable coverage, but are (by their very nature) counterproductive to free access to statutes: ‘A private market for legal resources is antithetical to equal justice.’^21^ Their sheer commercial success up to this point may be a symptom, not a solution, ‘of inadequate public access to legal information, even in the developed countries’.^22^

In addition to (commercial or non-profit) private initiatives, there are also international organisations that collect laws from around the world. However, each limits its efforts to a thematically defined subset, such as on food and agriculture (UN’s FAOLEX: 200 countries), intellectual property (WIPO Lex: 199 countries), labour, social security and related human rights (ILO’s NATLEX: 196 countries) or asylum laws (European Country of Origin Information Network, ECOI: 169 countries). Albeit useful for comparative legal research on specific topics,^23^ collections of this kind remain eclectic at best and do not provide broad-basic access to any country’s legislation.

Instead of trusting private or sectorally limited international initiatives to provide public access to legal statutes, national governments should be the ones to bear the ultimate ‘responsibility for adequately publishing [their] law in formats that are accessible and usable by the public’, that is, by offering ‘free, unrestricted online access to official, authenticated, and reliably preserved primary legal texts’.^24^ A comprehensive assessment of the current state of official legal databases around the world, however, is still lacking.

### C. Our Contribution to Empirically Testing Normative Aspirations

To close the gap, the present study surveys 204 polities to assess whether they provide access to statutory laws in adherence to a few basic criteria, ie being freely accessible (not requiring any costs or registration), searchable (regarding the legal acts’ titles and full text), reusable (allowing users to select and copy text, rather than just view scanned image files) and plausibly comprehensive (without exhibiting obvious gaps, which would be the case, for instance, if a database provided laws dating back only to the past five years).

The remainder of this article is organised along our research process. A first objective, we needed to create a list of all the official legal databases (OLD)^25^ from 204 countries and jurisdictions by searching the internet in a systematic manner. In a second step, we tested each legal database against a catalogue of five basic criteria, which we consider a ‘minimum viability’ standard, as defined in section 2. Using this database, we found that only 97 out of 204 countries (48%) fulfil basic criteria of access to statutory law. In addition, 25 countries (or 12%) do not seem to offer any official online access to statutes at all. These results show a clear geographical concentration, with Europe faring best when it comes to public access to statutory law; the quality of the database is also positively associated with advanced economic development and a population’s Internet usage (section 3). This has important consequences for the conduct of legal research, for the practical work of jurists and for the general public, which we discuss in section 4, before concluding our investigation.

## 2. Methods

### A. Scope of Data Collection

To analyse the current state of official legal databases on a global scale, the study investigated 204 polities. This sample includes all 193 member countries of the United Nations (UN), as well as 11 so-called *de facto states*^26^ whose claims for sovereignty remain without widespread recognition, but which usually feature similar official institutions as recognised states.^27^ Our survey focused on the national level (ie on federal rather than state laws in the case of federal countries) and on general statutes, meaning laws promulgated below the constitutional level but with a broad scope of application (ie broader than regulations for individual cases or narrow contexts such as bylaws, decrees or edicts).

This implies two inherent limitations that we wish to acknowledge at the outset. The first limitation is this study’s exclusion of laws below the country level, especially state laws in federal states. While some may be tempted to dismiss these ‘lower level’ laws (which are often superseded by hierarchically ‘higher’ laws) as less relevant, this may be warranted for some countries, but not others. For instance, most of what civil lawyers call ‘private law’ (contracts, torts, property, etc) is regulated in the United States almost exclusively at the state level, despite being vital for almost all business transactions. Therefore, we take no normative position on the (relative) importance of national laws as opposed to other legislative instruments, other than to suggest that national laws must be sufficiently relevant to justify the significant effort of top-level legislation. We do suggest, as a tentative proposition, that where access to national laws is fragmentary, one may not expect to find more at ‘lower’ levels. The reverse, however, need not be true: countries with excellent access to national laws need not provide the same quality of access in their constituent regions. This is an issue that we leave to further research, limiting ourselves to documenting a *lower bound* on public access to law.

The second limitation is this study’s exclusion of judicial case law, which commands binding authority in many countries. Yet, we know of no countries that do not *also* enact statutory laws, or countries that dismiss their statutory law as practically meaningless. Therefore, and again in the spirit of establishing *minimal* criteria, we expect even countries with a heavy emphasis on case law to still provide basic access to their statutory laws. We do not claim to document transparency of ‘the’ law across countries, but ‘merely’ access to statutory laws. While statutory laws are just one component of pretty much any legal system (‘law on the books’), they are an indispensable one nonetheless. Again, all that our study can (and will) provide is a lower bound estimate, without prejudice as to the relative importance of normative sources in any one jurisdiction, or even across jurisdictions.

### B. Data Collection Step 1: Identification

Two steps of data collection and analysis were necessary: first, to obtain the uniform resource locators (URLs) to each legal database (OLD); and second, to assess their functionality based on a uniform coding scheme.

Regarding the first step of data collection, URLs were collected manually since there was no central platform that harboured such links on a global scale. An earlier initiative that had aimed at presenting such a single platform, the *Center for Research Libraries’ Foreign Official Gazette Database*, was discontinued in 2007.^28^*Wikipedia* maintains a list of government gazettes, but it neither contains all countries nor URLs for each listed country, and many of the listed links are defunct or belong to private initiatives rather than official platforms.^29^ Another list, the EU-curated *N-Lex*, remains regionally bound to the European Union and its vicinity.^30^ The present study thus had to gather data from scratch by a targeted search regarding each country of the world. This search was conducted using Google as the most widely used search engine in most parts of the world. Despite known censorship issues, Google serves over 200 countries and territories,^31^ so we used it as the benchmark for the findability of legal databases.^32^ Using that search engine, we entered queries such as ‘[Country name] Legal Database’, ‘[Country name] Legislation’ or ‘[Country name] Official Gazette’. In case no official database could be found with these queries, we added the tag ‘site:*.tld’ to the query, with ‘tld’ denoting the top level domain of the respective country (eg ‘Saint Lucia laws site:*.lc’ or ‘Bolivia legislation site:*.bo’), or resorted to keywords such as ‘[Country name] Parliament’, ‘[Country name] Government’ or ‘[Country name] Ministry of Justice’, aiming to find second-order links to the legal databases via the websites of those respective organs. Where necessary, we also varied these keywords by language, as in ‘Angola Legislação’, ‘Colombia ley’ or ‘законы кыргызстана’ (zakony Kyrgyzstana). If this search strategy returned no results, we consulted curated sources as far as available—including the *Guide to Law Online*^33^ or *Wikipedia*’s list of government gazettes.

Once located, each country’s OLD was coded using the following variables: (i) country name and country codes from the International Organization for Standardization (ISO); (ii) URL to the database’s landing page; (iii) the referral website or Google keywords used to find it; and (iv) the date at which the URL was last accessed. In addition, we used the Internet Archive’s *Wayback Machine* (web.archive.org) to create a time-stamped snapshot of the OLD’s landing page for future reference, and documented its permalink (v).

### C. Data Collection Step 2: Coding

Having thus identified all national OLDs, the next step consisted of a fine-grained manual coding of their functionalities. We considered using a similar approach as the GODI project to ensure comparability,^34^ but this would have left out half the world’s countries due to a relatively high threshold of indexing. The present article thus used a simpler, less demanding coding scheme. We chose to build our approach upon the so-called FAIR data principles. They require that data be findable, accessible, interoperable and reusable in order to be deemed ‘open data’.^35^ In the legal context, this has been taken to mean that authoritative texts should be freely available, authentically official, comprehensive and reusable with the help of computer-supported techniques.^36^ Borrowing the term ‘minimum viability’, which is used for benchmarking new softwares in marketing research,^37^ we operationalise an analogous concept that we call *minimum viable official legal database*. An OLD is thus minimum viable if the given database fulfils the following five criteria, each of which was coded in a binary variable as 1 if the database exhibited the given criterion or 0 if absent:

(1) *Free availability—*Does the state provide an OLD for online access without requiring any registration, login or payment? To include as many countries as possible in our survey, we accepted the website of any official organ (such as the Parliament, the Ministry of Justice or the Presidency), even if it merely linked to a handful of legal documents. The idea is to acknowledge even nascent efforts at systematically collecting statutes through a single point of access. Drawing from the FAIR principles, we made a conscious choice to interpret ‘free’ not as merely cost-free (ie *gratis*), but also devoid of barriers (ie *libre*) which would force the user to leave whatever anonymity their browser affords them.^38^ If no *free* database in this sense could be found, the country was coded as 0 and dropped from further analysis. Otherwise, it was coded as 1, proceeding with the next four criteria.
(2) *Searchability (titles)*—Does the database provide a search function for users to find statutes based on matching keywords in their title? OLDs that were limited to browsing functionality (thereby requiring users to look for specific content) were coded as 0.^39^ To discount website search functions that return all kinds of documents and thereby clutter the results with extraneous content (eg if an OLD on a parliamentary website searched the whole website, including news items or biographies of Members of Parliaments, and not just statutes), we also coded such instances as 0. Otherwise, if a keyword search returned any and only statutory texts, we coded the OLD as 1 on this criterion.
(3) *Searchability (full text)*—Does the database provide a function to search within the laws’ full texts? Again, the function was expected to be dedicated exclusively to statutory texts (and not cover news items etc), otherwise it was coded as 0.
(4) *Reusability—*Can users select contents of statutes and copy them for further processing? If, for instance, the OLD offered merely scanned images in portable document format (.pdf),^40^ this criterion was coded as 0. This might correlate with criterion 3 (full text searchability), but needs not: some databases have full texts indexed for search but provide only limited options for accessing texts. Regardless of searchability, then, we coded this criterion as 1 where text selection and copying were possible and 0 where they were impossible without further processing, or even prohibited by technical means.
(5) *Plausible comprehensiveness*—Does the OLD purport or appear to be comprehensive? Given that we could not rely on benchmarks of how much statutory law exists in any given country, we were limited to a plausibility check: if an OLD claimed to be comprehensive and was not obviously limited (in time or topic), we coded this criterion with 1. We proceeded likewise where an OLD stated nothing about its coverage but seemed to be comprehensive given the country’s size and political activity. We tried to err on the side of caution and merely to filter out cases where an OLD plainly provided just a limited selection of statutes. These cases were coded as 0.

Taken together, the presence of all five criteria added up to a variable score of 5, which we considered the minimum viable public access to statutory law.^41^ Lower scores might be considered critical, with a score of 0 indicating the lack of any OLD.

Our coding thus used rather simple criteria with a straightforward cumulation of the total score. This is not to deny that legal databases could be assessed on more nuanced criteria and more sophisticated statistical metrics.^42^ For instance, one could ascertain the existence of metadata on whether a given statute is currently in force, when it entered into force or whether it has been amended (and, if so, by which other statute), accompanied by visualisations that illustrate patterns of textual change over time. Or one could examine whether the database offers an application programming interface (API) from which users can bulk-download statutes or run scripted programs on them, or whether the database provides structured links to court judgments invoking the respective statute or its individual provisions. Alternatively, one could add a further criterion regarding the availability of official English translations of the legal statutes (since English is the global *lingua franca*). Such features would greatly facilitate international legal accessibility, and we encountered positive exemplars, eg South Korea’s legal database. Yet, for our initial survey probing basic criteria for the existence of *any* online OLD at all, we chose to disregard such finer distinctions. Our dataset, however, is freely available for anyone to amend and enrich, as further research may necessitate.^43^

Using the above coding scheme, the actual coding was conducted manually by the primary author (AN-P) in at least three iterations (in December 2020 and in April and June/July 2021), while the secondary author (HH) verified about 35% of the codings, finding few mistakes or reasons for disagreement. These were discussed among the authors and jointly resolved.

## 3. Results

Using the coded dataset obtained from the methodology just described, we now proceed with a first descriptive analysis of our data (A) to identify basic patterns in geographic distribution and regional variation (B) and to examine how far our data are associated with economic development and Internet usage (C). To go into greater detail with a view to individual countries, we discuss specific examples of our coding outcomes in the Appendix, which also elaborates on cases where coding proved difficult or resulted in a total score of zero.

### A. Data Description

Our coding scheme resulted in a dataset of 204 rows and 18 columns, comprising 3672 values elicited for each of the 204 polities we studied. These values included, *inter alia*, the country names and country codes, the (original and archived) URLs to the OLDs, the path through which we found them, one binary variable for each of the criteria we defined and the total score for each country.

As a first descriptive observation, our four criteria were fulfilled, respectively, by 88% (free availability), 65% (title searchability), 50% (full text searchability), 78% (reusability) and 66% (plausible comprehensiveness) of countries. Considering the distribution of these criteria further, Table 1 offers an overview of all correlation coefficients (leaving out the first criterion of the mere existence of an OLD because it is not independent from the others).

**Table 1.** Correlation coefficients among the criteria with which the official legal databases (OLD) were assessed

	Searchability (titles)	Searchability (full texts)	Reusability	Comprehen­siveness
Searchability (titles)	1			
Searchability (full texts)	0.74	1		
Reusability	0.52	0.46	1	
Comprehensiveness	0.66	0.65	0.59	1

Every correlation was statistically significant (*p* < 0.05).

As Table 1 shows, there are no negative correlations among the criteria. A strong positive correlation existed, unsurprisingly, between the two aspects of searchability; that is, an OLD with searchable titles will likely feature full text search as well. The other correlations have effect sizes that are moderate, ie higher than 0.4 but lower than 0.7.^44^

### B. Geographic Distribution and Regional Variation

Moving from our five single criteria to the cumulative score for each country, we observe that less than half the world’s countries (97 out of 204, or 48%) provide a score of 5, ie minimum viable access to statutes. In contrast, other parts of the world (82 out of 204 jurisdictions, or 40%) do not meet our threshold of minimum viability, meaning that while they do offer free access to their laws, they either do not provide a search function, are not reusable or lack comprehensiveness. Finally, 25 countries (12%) do not seem to offer any OLD with free access to statutes at all. The detailed breakdown by country is shown in Table 2.

**Table 2.** Overview of our assessment of OLDs around the world, based on 204 polities, with scores ranging from 0 (no database at all) to 5 (minimum viable legal database)

Assessment	Score	*N*	Countries
Minimum viable legal database	5/5	97 polities (48%)	Albania, Algeria, Andorra, Antigua and Barbuda, Argentina, Australia, Austria, Bahrain, Bangladesh, Belarus, Belgium, Benin, Bolivia, Brazil, Bulgaria, Canada, Chile, Croatia, Cuba, Cyprus, Czech Republic, Denmark, El Salvador, Estonia, Finland, France, Gabon, Georgia, Germany, Greece, Grenada, Hungary, Iceland, India, Indonesia, Iran, Iraq, Ireland, Italy, Japan, Jordan, Kazakhstan, Kenya, Korea (ROK), Kyrgyzstan, Latvia, Liechtenstein, Lithuania, Luxembourg, Madagascar, Mali, Malta, Mexico, Moldova, Monaco, Mongolia, Myanmar, Namibia, Nauru, Netherlands, New Zealand, Nicaragua, Norway, Pakistan, Philippines, Poland, Portugal, Qatar, Romania, Russia, St Kitts and Nevis, San Marino, Serbia, Singapore, Slovakia, Slovenia, South Africa, Spain, Sweden, Switzerland, Taiwan, Tajikistan, Togo, Tonga, Transnistria, Trinidad and Tobago, Tunisia, Turkey, Turkish Republic of Northern Cyprus, Turkmenistan, Tuvalu, Ukraine, UK, USA, Uruguay, Uzbekistan, Vietnam
Below a threshold of minimum viability	4/5	21 polities (10%)	Afghanistan, Azerbaijan, Bahamas, Barbados, Bhutan, Bosnia and Herzegovina, Djibouti, Dominica, Donetsk, Fiji, Guyana, Israel, Jamaica, Lugansk, Montenegro, Peru, Seychelles, Sri Lanka, Thailand, United Arab Emirates, Tanzania
	3/5	26 polities (13%)	Abkhazia, Armenia, Brunei Darussalam, Cambodia, China, Congo, Costa Rica, Guatemala, Kosovo, North Macedonia, Maldives, Marshall Islands, Micronesia, Nagorno-Karabakh, Nepal, Oman, Palestine, Panama, Paraguay, Samoa, Sierra Leone, South Ossetia, Suriname, Syria, Timor-Leste, Zambia
	2/5	25 polities (12%)	Belize, Burkina Faso, Burundi, Colombia, Ethiopia, Ghana, Guinea, Guinea-Bissau, Honduras, Lebanon, Malaysia, Mauritania, Mauritius, Morocco, Mozambique, Niger, Rwanda, St Lucia, Saudi Arabia, Senegal, Somaliland, Swaziland, Uganda, Venezuela, Yemen
	1/5	10 polities (5%)	Cameroon, Ecuador, Kiribati, Laos, Lesotho, Libya, Malawi, Papua New Guinea, Solomon Islands, Sudan
No database found	0/5	25 polities (12%)	Angola, Botswana, Cape Verde, Central African Republic, Chad, Comoros, Côte d’Ivoire, Democratic Republic of the Congo, Dominican Republic, Egypt, Equatorial Guinea, Eritrea, Gambia, Haiti, Korea (DPRK), Kuwait, Liberia, Nigeria, Palau, St Vincent and the Grenadines, São Tomé and Príncipe, Somalia, South Sudan, Vanuatu, Zimbabwe

**Figure 1.**
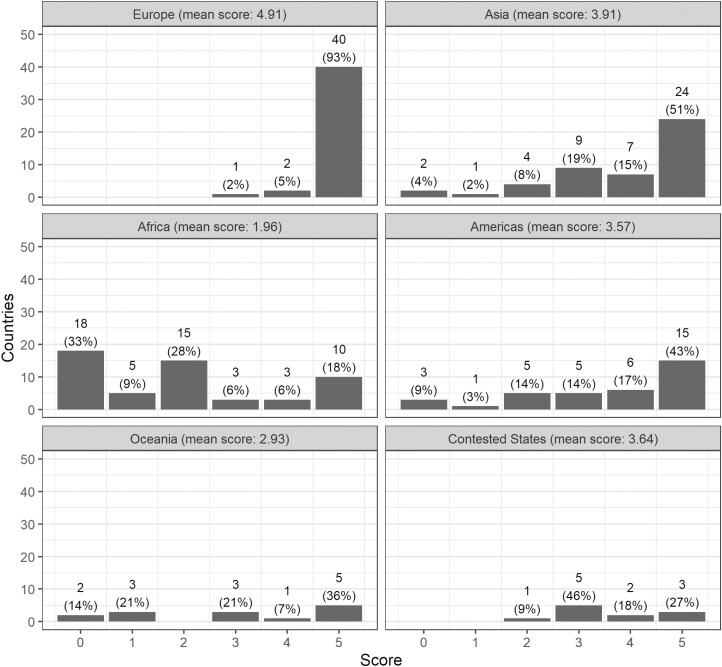
Discrete histogram of total scores for each country, grouped by continents. Contested *de facto* states are shown separately.

These data point to a regional concentration regarding access to statutes. Grouped by continents^45^ (Figure 1), Europe outperforms the other continents when it comes to providing access; most European countries attained the highest score, two obtained a score of 4 (Bosnia and Herzegovina, and Montenegro), while only one had Europe’s lowest score of 3 (North Macedonia). In contrast, the most prevalent score in Africa was 0, with only 10 (out of 55) countries providing a minimum viable OLD. In the Americas, Asia and Oceania, 5 was the most frequent score, but the distribution was much flatter than in Europe. Each of these continents features countries with a total score of 0. Interestingly, all of the contested *de facto* states were positioned at the higher bound of the score range, confirming that their state-building efforts are not to be underestimated despite the absence of widespread recognised sovereignty.^46^ The association between continent and a given OLD’s score was significant under a chi-square test, with χ^2^ (25, *N* = 204) = 114.29 and *p* < 0.001.

### C. Economic Development and Internet Usage

**Figure 2.**
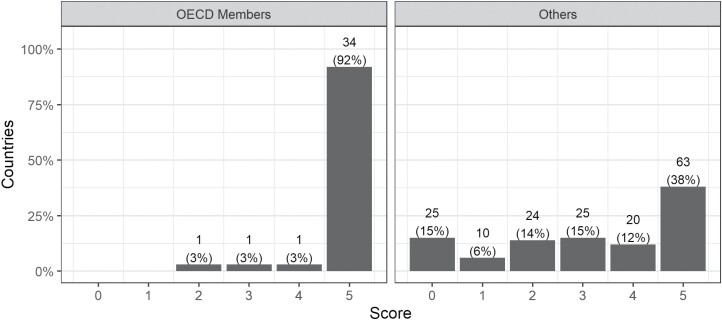
Discrete histogram of total scores for countries with and without membership in OECD.

Moreover, the level of a country’s economic development and the provision of a minimum viable OLD are significantly associated. A chi-square test yields χ^2^ (5, *N* = 204) = 35.917, with *p* < 0.001. Almost all states with membership of the Organisation for Economic Co-operation and Development (OECD, an international organisation comprising highly advanced economies) obtained a score of 5; the only exceptions were Israel (score of 4), Costa Rica (score of 3) and Colombia (score of 2). While many non-OECD members likewise provide a minimum viable OLD, the distribution of scores is much flatter (see Figure 2).

**Figure 3.**
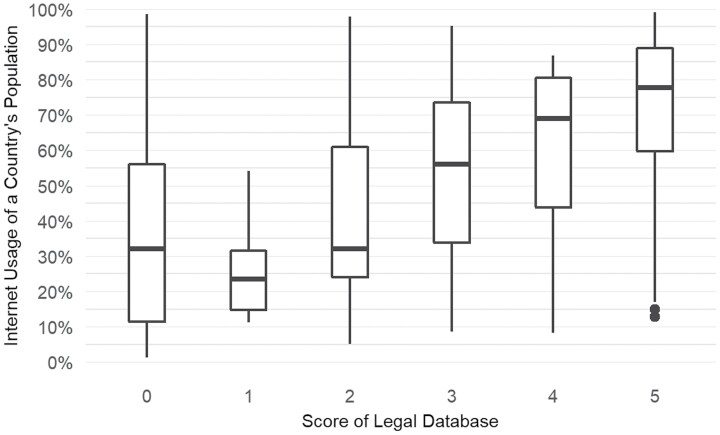
Boxplot showing the association between the number of individuals using the Internet (% of a country’s population) and the score attained by that country’s official legal database.^48^

In addition, and perhaps unsurprisingly, a given population’s overall Internet usage (based on data from the World Bank)^47^ significantly relates to the respective OLD score, according to an analysis of variance test (*F*(5, 186) = 16.12, *p* < 0.001). Figure 3 illustrates the associations.

## 4. Discussion

### A. Summary and Limitations

**Figure 4.**
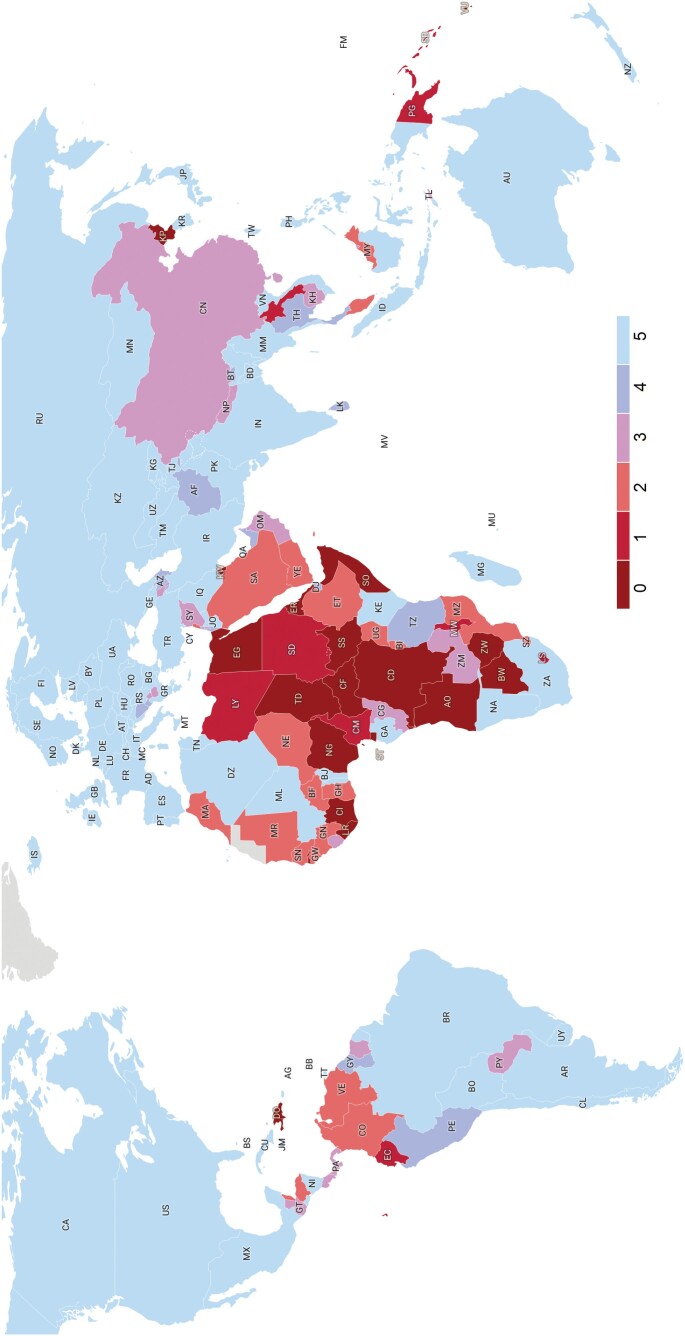
World map with colour codings based on the total score for each country. Visualisation created as a chloropleth map in *Datawrapper* (app.datawrapper.de).

Our survey of the global state of public access to statutory law found that 97 out of 204 countries, or slightly less than half of the world’s sovereign jurisdictions (48%), offer a minimum viable legal database for anyone to use for free. Twenty-five countries (12%) do not seem to provide any online access to their statutory laws at all. The remaining 82 jurisdictions (40%) do offer OLDs, but of a quality below minimum viability, meaning that they lack search functionalities (in titles or in the full text of statutes), they do not allow users to easily reuse the legal texts or their databases are clearly less than comprehensive. Figure 4 illustrates our findings.

Our results are subject to various limitations. In a transnational data collection effort such as this, erroneous omissions and minor misclassifications can hardly be ruled out. We tried to hedge against such mistakes through, first, a systematic sampling and coding approach with high transparency so as to render its methods reproducible. This is in contrast to websites that collect links to legal datasets in a ‘crowdsourcing’ manner (such as *Wikipedia*’s list of gazettes) without describing their method of ascertaining the URLs or guaranteeing a uniform approach. A second approach to mitigate the limitations of this study lies in the public sharing of the resulting dataset, which enables anyone to reuse the coding scheme, to add their own findings or to amend and update the data.

Another limitation lies in the fact that the results merely offer a snapshot as of mid-2021. To counteract the distorting effect of merely considering one point in time, the survey was conducted in three iterations, in December 2020, and April and June/July 2021. In that way, OLDs were only coded as non-existent when their URL repeatedly returned error messages over several months. In addition, we archived the landing pages of OLDs in the Internet Archive’s *Wayback Machine*, so as to document how the databases appeared at the time we accessed them. Nevertheless, despite our best attempts at transparency and replicability, we cannot guarantee flawless (or even timeless) results, but merely hope to offer a useful first basis for assessing the state of legal accessibility across the globe.

### B. Policy Implications

Our finding that more than half the world’s jurisdictions do not even provide a minimum viable OLD may help to highlight negative impacts on at least three groups of stakeholders: (comparative) legal researchers, practitioners of law and the general public.

First, researchers engaged in comparison of laws face difficulties in accessing even the ‘law on the books’ of many countries, despite the global reach of the Internet. The fact that statutory laws from the Global South are not as readily available as those from more developed economies will bias our understanding of the law towards a select group of advanced economies. There is indeed an impression that leading journals of comparative law mainly publish case studies regionally concentrated in the Global North.^49^ The lack of reliable availability in the form of minimum viable OLDs likely contributes to this imbalance. Research with greater regional inclusivity remains an exception, with many such studies focusing exclusively on the *constitutional* level,^50^ rather than other legal statutes. A cost-efficient approach to comparative law *below* the constitutional level will still have to leave out, and thus indirectly contribute to making invisible, the Global South.

This widespread inaccessibility might have repercussions not only for scholars of comparative law, but also for students of international law, since legal institutions such as ‘general principles of international law’ or ‘customary international law’ often derive from doctrines found *in foro domestico*, ie domestic legal statutes.^51^ As long as statutes from many countries are unavailable, international harmonisation will be limited to just a few (economically advanced) states, from which international lawyers infer ‘general principles’ that are then considered binding for the entire international community.^52^ Broader access to national statutes would offer a more satisfying solution in terms of sample size, global ethics and legal soundness. In legal research, well-funded researchers have been able to offer more comprehensive surveys of municipal laws by pursuing ‘leximetric’ studies that cover up to 117 countries.^53^ Yet in order to do so, they need to consult local jurists from many of these countries,^54^ requiring vast amounts of research funding.^55^ Such costs could be decreased considerably through greater commitments to open data and open government principles. Authoritative legal texts should be free for any jurist, researcher, citizen, migrant or anyone from all around the world.

This segues into the second group of stakeholders affected by a lack of minimum viable access to statutes, namely local jurists, attorneys and other legal practitioners. They are the ones who most directly rely on authoritative, up-to-date versions of legal statutes for their work. But in at least half the world, jurists need to overcome steep hurdles when ascertaining the current state of local laws. Given the lack of a minimum viable OLD, determining the legal status quo will always be costlier than if there was an officially mandated, free-to-use database with the authoritative texts of all statutes. Such platforms reduce not only the time needed (thus increasing the efficiency of lawyering), but also monetary costs, insofar as legal offices no longer need to rely on commercial providers or even to keep printed versions of legal texts up-to-date manually.

Third, and beyond the domain of legal scholarship and practice, the lack of minimum viable access to statutes also means that a great number of the wider populace faces increased insecurity when it comes to their legal situation—which disproportionately affects minorities and the economically poor.^56^ While access to statutory texts does not imply instant legal clarity (since statutes are just one component of any legal system, as we pointed out earlier, and because legal texts still require skilful interpretation),^57^ a lack of access certainly makes matters worse by enforcing exclusive reliance on professional elites. Citizens who lack access to statutory law often face a deficient position in power-asymmetric contexts, be it in relation to their landlords, their employers or their local bureaucracies. Without knowledge of one’s rights, there are high barriers to articulating one’s claims.^58^ Being stripped of one’s ‘legal voice’,^59^ unfavourable outcomes are likely.^60^ This state of legal disenfranchisement affects not only individual lives, but also society as a whole. Legal uncertainty radiates to the economic sector and others, eg by deterring potential investors from abroad.^61^ A general inaccessibility of law thus erodes societal well-being on individual and collective levels.

This is not to say that adequate access to statutory law is a panacea, or even achieved by just dumping a country’s statutes into an OLD. In a fundamental sense, access to statutes is not just about having them at one’s disposal in the digital realm,^62^ but also entails the ability of citizens to adequately understand the provisions^63^ and to know how to navigate the legal system, including in financial, procedural and social terms.^64^ In that sense, availability of texts is a *necessary* but by no means a *sufficient* condition for access to statutory law.^65^ Its being a necessary condition, however, is already sufficient motivation for continued research in this domain.

### C. Future Paths

Despite the current costs of legal inaccessibility, the future may hold improvements. Large-scale efforts drawing on technological advances and easy-to-use standard templates may address this issue. The existing open government frameworks serve as harbingers of this development in legal information infrastructures. Rather than aiming at the mere minimum we envisaged here, practical solutions may harness current research on legal information technology so as to establish digital corpora with well-structured laws and annotated metadata, such as standardised XML schemes.^66^ Such datasets would then employ

machine-readable formats, assigning standardised metadata to the published documents and datasets, providing both programmable and bulk access to documents and data, explicitly publishing licences which apply to them in a machine-readable format, and introducing a centralised portal enabling retrieval and browsing of open data sets from a single source.^67^

That way, OLDs around the world might not only achieve minimum viability, but attain advanced functionalities worthy of data driven digitised societies. They would resemble what open data advocates call findable, accessible, interoperable and reusable, or FAIR. Following this lofty path of FAIR-ness could result in the long-term goal of a single ‘legal information system capable of supporting the legislation of all countries’ around the world,^68^ or a global point of access to legal databases comprising all jurisdictions at once.^69^

Such comprehensive databases could enable research that expands upon recent studies on ‘algorithmic decision-making’ in law^70^ or on the use of machine-learning technologies to trace semantic change in legal language.^71^ Conducting such research on a global, rather than a national, scale would be in tune with legal issues that increasingly transcend national borders and require global solution approaches (eg financial markets, climate change, international security). But as long as such unitary access points are lacking, our research suggests a more pragmatic approach.

By combining the present dataset with other parameters, researchers can use our data as control variables in a wide variety of cross-country research projects, enabling them to study effects of social change on law or *vice versa*, and to provide a fuller description of law-related surveys of the world’s countries. Furthermore, conspicuous patterns in the dataset can serve as starting points for other kinds of in-depth explorations, such as those involving former colonial ties.^72^ For example, a number of former Portuguese colonies—Angola, Cape Verde, Equatorial Guinea, and São Tomé and Príncipe—do not offer any OLD at all. Might this result from Portuguese law still being (partly) in force in some of these countries?^73^ Does it mean that they have not prioritised establishing their own OLD, even if they thereby prolong the ‘inheritance’ of their colonial legacy?^74^

In addition, legal practitioners and data brokers might use the URLs we collected in our dataset as a ‘living document’ from which to access legal databases around the world. Others may use it to pressure governments to do more about their rule of law and legal access. Scholars might use the FAIR approach as a template to assess how other stately branches and organs fare, such as the judiciary or whether parliamentary discussions are globally accessible as open data. The progress (or stagnation) towards ‘open government’ is thus made quantifiable, claimable and exactable. Our empirical finding that the world is not yet ready for ubiquitous access to statutory law does not devalue normative arguments for why legal access is paramount for society at large—just as it does not shake our conviction that, ultimately, anyone across the globe should be able to read the statutes of any place without incurring prohibitive costs.

## Appendix: Explanation of Codings and Doubtful Cases

This appendix documents reasons for individual codings, as well as cases where coding countries proved difficult. This includes cases where our coding resulted in an overall score of 0, which we double-checked to make sure we consistently erred on the side of caution.

### A. Criterion 1: Free Availability

Given our predefined limitation to *official* databases, we disregarded any *private* initiatives (commercial or not). For instance, privately owned platforms in Azerbaijan (*legalacts.az*), the Democratic Republic of Congo (*leganet.cd*) or Ethiopia (*ethiopialaw.com*) were not considered OLDs, even when they seemed up-to-date, comprehensive and equipped with elaborate technical functionality.

There were 25 cases where we could not find any official, freely accessible legal database. Some of these may not offer any kind of public access to law at all (eg Comoros, Eritrea, Equatorial Guinea, Nigeria, North Korea), while others suffered from a technical instability, returning error code 404 (‘page not found’) on multiple occasions from late 2020 to mid-2021 (eg Democratic Republic of Congo, Gambia). São Tomé and Príncipe’s database was ‘under construction’ during that same whole period, while Somalia’s remained in a constant state of ‘coming soon’. Angola’s web portal for laws merely led to a login form without any other content (same as Côte d’Ivoire), and Guatemala’s required a registration without which one could see only non-downloadable PDF previews. Chad’s official journal simply remained empty. Cabo Verde listed various titles of laws with specific links, but every one of these links led to error pages (similar to Venezuela); South Sudan enumerated the titles of some legal acts, but these did not link to their contents. Zimbabwe’s database only exhibited a dysfunctional input screen that never led to any results at all. All of these cases were coded with 0 on the first criterion, and therefore 0 overall.

### B. *Criterion 2: Searchability (Titles)*

Given our predefined criterion of a keyword search rather than mere browsing or numerical inputs to retrieve specific documents by number or date, we had to code 73 countries as 0. Some countries did not offer a query function, but only allowed users to browse through laws by topics (eg Armenia, United Arab Emirates), in alphabetical order (eg Antigua and Barbuda, Grenada) or chronologically by time of a legal act’s creation (eg Ecuador, North Macedonia, Zambia). Others provided only a general website search that cluttered results with extraneous information (eg Ethiopia, Mauritius, Mozambique, Paraguay, Timor-Leste), or a dysfunctional search bar that never yielded any results (eg Mauritania). In a few cases, the search function was limited to specific legislative periods (eg Micronesia, Morocco). All of these cases were coded as 0.

### C. *Criterion 3: Searchability (Full Texts)*

Even databases that allowed to search the titles of statutes did not always enable full text search (eg Barbados, Montenegro, Panama, Tanzania, Thailand). These instances were coded as 0 on our third criterion. In addition, we again coded 0 for databases with dysfunctional search bars or those that comprised all kinds of documents other than statutes. This meant that a total of 103 countries had to be coded as 0 on this criterion.

### D. *Criterion 4: Reusability*

The fourth criterion required users to be able to retrieve, store and reuse (ie select, highlight and copy) the statutory texts from an OLD. This was precluded where statutes were merely presented as scanned images without having been converted into machine-readable text (eg Burundi, Cambodia, Cameroon, Laos, Libya, Papua New Guinea, Solomon Islands), sometimes with such low image quality that any further processing (even with advanced OCR technology^75^) would be prohibitively difficult. In some countries, we observed differential treatment regarding reusability, such as older statutes failing to fulfil our criterion, while newer statutes did (eg Congo, Dominica, Sierra Leone, Suriname). We coded such cases as 1 on this criterion to acknowledge the recent efforts towards reusability. The same approach was taken with regard to Turkey, where statutes seemed to be provided in copyable text format, while other kinds of documents (such as presidential decrees) were only offered as scanned images. A special problem of reusability was posed by Monaco, whose database allows users to read statutes within their browser, but only section by section. There was thus no straightforward way to download the full texts of laws. Despite this major inconvenience, we nevertheless coded Monaco with 1 because it did allow readers to ‘reuse’ the legal statutes in the broad sense of the word defined in section 2. Overall, we coded just 46 OLDs as 0 on this criterion.

### E. *Criterion 5: Plausible Comprehensiveness*

The fifth and final criterion could not be fulfilled wherever a polity listed only *recent* legal acts (eg Belize dates back only to 2015, Ecuador to 2009, Kiribati to 2016, Laos to 2004, Lesotho to 2020, Mauritius to 2009, Mozambique to 2006), or conversely *no* recent acts (eg Ghana lists only until 2017, Solomon Islands stopped in 2018, Malaysia seemed to offer full texts only to older laws, but not to the newest ones). In some instances, the OLD did not cover intermittent periods (eg the years 2001–10 and 2012–15 are missing from Colombia’s list of gazettes), in others they provided only partial lists of statutes (Panama lists just 42 documents, Sudan 22, Niger 13, Ethiopia 12, Malawi 5, Guinea-Bissau 4, Swaziland 3). In all such cases, while our inclusive definition of the first criterion did confirm an OLD to be present, it was still coded as 0 on the fifth criterion. A difficult case was the United States, where comprehensiveness was not easy to assess because there is no single point of access to the OLD, but rather a collection of different (albeit official) websites. Since the ‘US Code’ is available from a Government Publishing Office (in reusable formats including XML), we did code comprehensiveness with 1, but remain doubtful about the effective accessibility of statutes in the United States to untrained laypeople.

## Data Availability

The data that support the findings of this study are openly available on *Zenodo* at https://doi.org/10.5281/zenodo.6992389.

## Footnotes

1 Leesi Ebenezer Mitee, ‘The Right of Public Access to Legal Information: A Proposal for Its Universal Recognition as a Human Right’ (2017) 18 German Law Journal 1429, 1429.

2 Thomas R Bruce, ‘Public Legal Information: Focus and Future’ (2000) 1 JILT.

3 Andrew J Martineau, ‘Reinforcing the Infrastructure of Legal Research through Court-Authored Metadata’ (2020) 112 Law Library Journal 5, 16.

4 Christoph Engel, ‘Learning the Law’ (2008) 4 Journal of Institutional Economics 275.

5 Bruce (n 2).

6 NW Barber, ‘Must Legalistic Conceptions of the Rule of Law Have a Social Dimension?’ (2004) 17 Ratio Juris 474.

7 Ginevra Peruginelli, ‘Law Belongs to the People: Access to Law and Justice’ (2016) 16 Legal Information Management 107, 107.

8 The Free Access to Law Movement, ‘Declaration on Free Access to Law’ (FALM 2002) <www.web.archive.org/web/20220124152855/falm.info/declaration> accessed 30 July 2022.

9 Janneke Gerards, ‘A Right of Access to Law—or Rather a Right of Legality and Legal Aid?’ in Andreas von Arnauld, Kerstin von der Decken and Mart Susi (eds), *The Cambridge Handbook of New Human Rights: Recognition, Novelty, Rhetoric* (CUP 2020) 556–7.

10 Simon Rice, ‘Bentham Redux: Examining a Right of Access to Law’ in von Arnauld, von der Decken and Susi (n 9); Gerards (n 9).

11 European Court of Human Rights, *Sunday Times v The United Kingdom (No 1)* App no 6538/74, Judgment, para 49.

12 Rodrigo Sandoval-Almazan and J Ramon Gil-Garcia, ‘Understanding E-Justice and Open Justice through the Assessment of Judicial Websites: Toward a Conceptual Framework’ (2020) 38 Social Science Computer Review 334; Marko Marković and Stevan Gostojić, ‘Open Judicial Data: A Comparative Analysis’ (2020) 38 Social Science Computer Review 295; Hanjo Hamann, ‘The German Federal Courts Dataset 1950–2019: From Paper Archives to Linked Open Data’ (2019) 16 Journal of Empirical Legal Studies 671; Hanjo Hamann, ‘Der blinde Fleck der deutschen Rechtswissenschaft—Zur digitalen Verfügbarkeit instanzgerichtlicher Rechtsprechung’ (2021) 76 Juristenzeitung 656.

13 GODI, ‘National Laws’ (Global Open Data Index, 2016) <www.index.okfn.org/dataset/law> accessed 5 May 2021.

14 Zachary Elkins and others, ‘Constitute: The World’s Constitutions to Read, Search, and Compare’ (2014) 27–28 Journal of Web Semantics 10.

15 Daniel Hürlimann and Hanjo Hamann, ‘Inhalt des BGBl öffentlich: Erstmals offene Gesetze?’ (*Legal Tribune Online*, 2018) <www.lto.de/persistent/a_id/32689> accessed 5 May 2021.

16 Graham Greenleaf, ‘Free Access to Legal Information, LIIs, and the Free Access to Law Movement’ in Richard A Danner and Jules Winterton (eds), *The IALL International Handbook of Legal Information Management* (Routledge 2016); Philip Chung, Graham Greenleaf and Andrew Mowbray, ‘Models for a Global System for Free Access to Legal Information: The WorldLII Approach’ in Stephan Weth and Samuel van Oostrom (eds), *Festschrift für Maximilian Herberger* (Juris 2016) 193.

17 Martineau (n 3) 12; Susan Nevelow Mart, ‘The Algorithm as a Human Artifact: Implications for Legal [Re]Search’ (2017) 109 Law Library Journal 387.

18 This is especially true if their method of acquiring the legal texts is intransparent, but it even applies to the deliberately transparent *OKFN* project in Germany: it merely covers federal law, no state laws.

19 There is indeed evidence of fragmentary collection and year-long delays on WorldLII: for instance, Seychelles’ legislation list was last updated in 2005 and only contains 10 documents; the section for Samoa impressively dates back to 1920, but the most recent act listed is from 2012 (as of mid-2021).

20 WorldLII, ‘Disclaimers of Liability’ <www.worldlii.org/worldlii/disclaimers.html> accessed 5 May 2021.

21 Andrew Higgins, ‘What Price Are We Willing to Pay for the Dream of Equal Justice?’ (2022) 42 Oxford Journal of Legal Studies 325, 325.

22 Mitee (n 1) 1432.

23 Jody Heymann and others, ‘Preventing Immigration Detention of Children: A Comparative Study of Laws in 150 Countries’ (2021) 26 International Journal of Human Rights 591.

24 Leslie Street and David Hansen, ‘Who Owns the Law? Why We Must Restore Public Ownership of Legal Publishing’ (2020) 26 Journal of Intellectual Property Law 205, 206.

25 An official legal database may be the digitised version of the government gazette, but it need not be. For example, in Austria, the online database *Rechtsinformationssystem* is not the same as the gazette, the *Bundesgesetzblatt*; the database collects legal information with richer metadata and more granular presentation than the gazette. In Gabon, on the other hand, the online version of the gazette (or *Journal Officiel*) simultaneously serves as the online platform that offers free access to legal statutes.

26 These are Abkhazia, Donetsk, Kosovo, Lugansk, Nagorno-Karabakh, Palestine, Somaliland, South Ossetia, Taiwan, Transnistria and the Turkish Republic of Northern Cyprus.

27 Andreas Pacher, ‘The Diplomacy of Post-Soviet De Facto States’ (2019) 33 International Relations 563.

28 Center for Research Libraries, ‘Foreign Official Gazette Database’ (Global Resources Network 2007) <www-apps.crl.edu/fog> accessed 27 July 2021.

29 Wikipedia, ‘List of Government Gazettes’ (2021) <www.en.wikipedia.org/w/index.php?title=List_of_government_gazettes&oldid=1021458124> accessed 5 May 2021.

30 Doris Liebwald, ‘Interfacing between Different Legal Systems Using the Examples of N-Lex and EUR-Lex’ in Günther Grewendorf and Monika Rathert (eds), *Formal Linguistics and Law* (De Gruyter Mouton 2009) 257.

31 David Goldman, ‘Google: The Reluctant Censor of the Internet’ (*CNN*, 4 January 2015) <www.money.cnn.com/2015/01/04/technology/google-censorship/index.html> accessed 20 September 2021.

32 Given the aforementioned censorship issues, we cannot rule out that we found more OLDs by performing our search from servers within Europe than users on other continents might. Less likely, but still possible, is the reverse: we may have missed (very few) OLDs through *Google* that exist but are inaccessible from Europe.

33 Library of Congress, ‘Nations of the World’ (*Guide to Law Online*, 2020) <www.loc.gov/law/help/guide.php> accessed 5 May 2021.

34 cf Marković and Gostojić (n 12).

35 Mark D Wilkinson and others, ‘The FAIR Guiding Principles for Scientific Data Management and Stewardship’ (2016) 3 Scientific Data 160018.

36 Corinna Coupette and Andreas Martin Fleckner, ‘Quantitative Rechtswissenschaft’ (2018) 73 Juristenzeitung 379.

37 Valentina Lenarduzzi and Davide Taibi, ‘MVP Explained: A Systematic Mapping Study on the Definitions of Minimal Viable Product’, 2016 42th Euromicro Conference on Software Engineering and Advanced Applications (SEAA) (2016) 112.

38 On these competing interpretations of ‘free’, see Peter Suber, ‘Gratis and Libre Open Access’ *SPARC Open Access Newsletter* (2 August 2008) <www.nrs.harvard.edu/urn-3:HUL.InstRepos:4322580> accessed 20 September 2021.

39 An argument might be made that browsable collections *can* be more accessible (if arranged logically by topics covering all documents) than a mere text dump with a non-browsable interface but full-text search. While this is theoretically true, we did not encounter non-searchable OLDs that offered a sufficiently well-maintained ontology so that every document would be reachable by systematic browsing.

40 Technically, text from images that were scanned and saved into.pdf files can be selected and copied once these images are converted into machine-readable text via a process called ‘optical character recognition’ (OCR). This process is liable to recognition errors and typographic obstacles such as line breaks and hyphenation, which would often require manual correction. Following our lenient approach, we solely assessed whether any text could be selected and copied at all, regardless of the use and reliability of OCR processes in getting there.

41 As one reader helpfully pointed out, future studies might investigate the quality and extent of metadata provided with OLDs (eg information about the status of a statute, ie whether it was repealed or amended) and code for a taxonomy of such metadata. Also, a sixth criterion might be established as ‘republication rights’, and OLDs could be coded for whether they enable downstream authentication (eg by providing digital signatures that could help to verify the authoritativeness of a published statute). Given our explained ‘minimum viability’ aspiration, we have to leave such ideas to future research.

42 eg Sandoval-Almazan and Gil-Garcia (n 12).

43 See the data availability statement at the end of this paper.

44 The interpretation of the effect sizes as ‘moderate’ or ‘strong’ is based on Christine P Dancey and John Reidy, *Statistics without Maths for Psychology* (Pearson Education 2007) 176.

45 The attribution of the respective continent is based on the UN Statistical Division’s Region Names.

46 Magdalena Dembinska and Aurélie Campana, ‘Frozen Conflicts and Internal Dynamics of De Facto States: Perspectives and Directions for Research’ (2017) 19 International Studies Review 254.

47 International Telecommunication Union, ‘Individuals Using the Internet (% of Population) | Data’ (World Bank 2020) <www.data.worldbank.org/indicator/IT.NET.USER.ZS> accessed 6 October 2021.

48 As a technical note, the plot was generated with the programming language R, using the *ggplot2* package. Accordingly, ‘the lower and upper hinges correspond to the first and third quartiles (the 25th and 75th percentiles)’ and ‘the upper whisker extends from the hinge to the largest value no further than 1.5 * IQR from the hinge (where IQR is the inter-quartile range, or distance between the first and third quartiles). The lower whisker extends from the hinge to the smallest value at most 1.5 * IQR of the hinge. Data beyond the end of the whiskers are called ‘outlying’ points and are plotted individually [as dots].’ See Hadley Wickham and others, ‘ggplot2: Create Elegant Data Visualisations Using the Grammar of Graphics’, 67 <www.CRAN.R-project.org/package=ggplot2> accessed 8 August 2022.

49 eg Pierre Legrand, ‘European Legal Systems Are Not Converging’ (1996) 45 International & Comparative Law Quarterly 52; Robert A Kagan, ‘Globalization and Legal Change: The “Americanization” of European Law?’ (2007) 1 Regulation & Governance 99; Mathias M Siems, ‘Convergence in Corporate Governance: A Leximetric Approach’ (2009) 35 Journal of Corporation Law 729; Ivana Isailovic, ‘Same Sex but Not the Same: Same-Sex Marriage in the United States and France and the Universalist Narrative’ (2018) 66 American Journal of Comparative Law 267; Paul F Scott, ‘Passports, the Right to Travel, and National Security in the Commonwealth’ (2020) 69 International & Comparative Law Quarterly 365; Paula Giliker, ‘Codification, Consolidation, Restatement? How Best to Systemise the Modern Law of Tort’ (2021) 70 International & Comparative Law Quarterly 271.

50 eg Tom Ginsburg, Svitlana Chernykh and Zachary Elkins, ‘Commitment and Diffusion: How and Why National Constitutions Incorporate International Law’ (2008) 2008 University of Illinois Law Review 201; Emanuela Carbonara and others, ‘Agency Culture, Constitutional Provisions and Entrepreneurship: A Cross-Country Analysis’ (2018) 27 Industrial and Corporate Change 507; Petra Stykow, ‘The Devil in the Details: Constitutional Regime Types in Post-Soviet Eurasia’ (2019) 35 Post-Soviet Affairs 122; Tofigh Maboudi, ‘Participation, Inclusion, and the Democratic Content of Constitutions’ (2020) 55 Studies in Comparative International Development 48.

51 Craig Eggett, ‘The Role of Principles and General Principles in the “Constitutional Processes” of International Law’ (2019) 66 Netherlands International Law Review 197, 208.

52 Jaye Ellis, ‘General Principles and Comparative Law’ (2011) 22 European Journal of International Law 949.

53 Zoe Adams and others, ‘The CBR-LRI Dataset: Methods, Properties and Potential of Leximetric Coding of Labour Laws’ (2017) 33 International Journal of Comparative Labour Law and Industrial Relations 59.

54 Priya P Lele and Mathias M Siems, ‘Shareholder Protection: A Leximetric Approach’ (2007) 7 Journal of Corporate Law Studies 17, 25; Adams and others (n 53) 72.

55 See the Repository of the University of Cambridge, ‘CBR Leximetric Datasets’ <www.repository.cam.ac.uk/handle/1810/256566> accessed on 11 June 2021, listing two grants from the Economic and Social Research Council (ESRC) which, in total, provided funds of £914,303, alongside five additional grants.

56 Siri Gloppen and Fidelis Edge Kanyongolo, ‘Courts and the Poor in Malawi: Economic Marginalization, Vulnerability, and the Law’ (2007) 5 International Journal of Constitutional Law 258; Chimaraoke Izugbara and others, ‘Regional Legal and Policy Instruments for Addressing LGBT Exclusion in Africa’ (2020) 28 Sexual and Reproductive Health Matters 1698905.

57 Jeremy Waldron, ‘The Rule of Law in Contemporary Liberal Theory’ (1989) 2 Ratio Juris 79; Jeremy Waldron, ‘The Rule of Law and the Importance of Procedure’ (2011) 50 Nomos 3.

58 Nicole Stybnarova, ‘Tokenizing and Articulating Protection of Women in Migration Law: Strategies of Exclusion in Contemporary Europe and the Nineteenth-Century USA’ (2022) 31 Social & Legal Studies 309.

59 Gloppen and Kanyongolo (n 56).

60 Aude Lejeune and Julie Ringelheim, ‘Workers with Disabilities between Legal Changes and Persisting Exclusion: How Contradictory Rights Shape Legal Mobilization’ (2019) 53 Law & Society Review 983.

61 Cheng Bian, *National Security Review of Foreign Investment: A Comparative Legal Analysis of China, the United States and the European Union* (Routledge 2020); Mehmet Nasih Tag, ‘Judicial Institutions of Property Rights Protection and Foreign Direct Investment Inflows’ (2021) 65 International Review of Law and Economics 105975.

62 M Ethan Katsh and Orna Rabinovich-Einy, *Digital Justice: Technology and the Internet of Disputes* (OUP 2017).

63 Gerards (n 9) 577.

64 Deborah L Rhode, *Access to Justice* (OUP 2004).

65 Connie Chang, ‘Improving Access to Free Online Legal Information through Universal Design: User Personas, User Journeys, a Proposal, and a Prototype’ (2021) 40 Legal Reference Services Quarterly 199.

66 Martineau (n 3) 24–6; XML (Extensible Markup Language) documents structure text by introducing standardised labels for recurring text elements. This reflects the logical structure of these texts, thereby allowing to extract complex relationships such as informational hierarchies or standardised controlled vocabularies. XML thus encodes documents into database-like structures so that specific information can be retrieved computationally; cf Ron Dolin, ‘XML in Law: The Role of Standards in Legal Informatics’ in Daniel Martin Katz, Ron Dolin and Michael J Bommarito (eds), *Legal Informatics* (CUP 2021) 61; for an early example of how legal statutes could be presented as XML files, see Andrea Marchetti and others, ‘Using XML as a Means to Access Legislative Documents: Italian and Foreign Experiences’ (2002) 10 ACM SIGAPP Applied Computing Review 54.

67 Marković and Gostojić (n 12) 295.

68 Michalis Avgerinos Loutsaris and Yannis Charalabidis, ‘Legal Informatics from the Aspect of Interoperability: A Review of Systems, Tools and Ontologies’ in *Proceedings of the 13th International Conference on Theory and Practice of Electronic Governance* (Association for Computing Machinery 2020) 732.

69 Martineau (n 3) 10.

70 Rebecca Williams, ‘Rethinking Administrative Law for Algorithmic Decision Making’ (2022) 42 Oxford Journal of Legal Studies 468.

71 eg Mitra Bokaei Hosseini and others, ‘Analyzing Privacy Policies through Syntax-Driven Semantic Analysis of Information Types’ (2021) 138 Information and Software Technology 106608; Chaojun Xiao and others, ‘Lawformer: A Pre-trained Language Model for Chinese Legal Long Documents’ (2021) 2 AI Open 79; Alex Schwartz, ‘The Changing Concepts of the Constitution’ (2022) 42 Oxford Journal of Legal Studies 758.

72 Daniel M Klerman and others, ‘Legal Origin or Colonial History?’ (2011) 3 Journal of Legal Analysis 379.

73 See Michael Bogdan, ‘The Law of the Republic of Cape Verde after 25 Years of Independence’ (2000) 44 Journal of African Law 86, 93; Fernando Dias Simões, ‘Portuguese-Speaking Africa and the Lusophone Legal System: All in the Family?’ (2017) 76 African Studies 86.

74 Sandra Fullerton Joireman, ‘Inherited Legal Systems and Effective Rule of Law: Africa and the Colonial Legacy’ (2001) 39 Journal of Modern African Studies 571.

75 Above n 40.

## Notes

The authors thank Mathias Siems, Paola Lopez and Felix Zopf for helpful comments on an earlier version of the article, as well as editors and anonymous reviewers from the *Oxford Journal of Legal Studies* and the *Journal of Empirical Legal Studies* for their valuable time and constructive input. We dedicate this paper to the memory of Professor Daniel Hürlimann (1985–2022), a German-speaking pioneer of open access to law, and a passionate advocate for judicial transparency, who contributed to various international reports on the state of open science; cf Anna Severin and others, ‘Discipline-Specific Open Access Publishing Practices and Barriers to Change: An Evidence-Based Review’ (2020) 7 F1000Research 1925; Swiss Academies of Arts and Sciences and others, ‘Open Science in Switzerland: Opportunities and Challenges’ (2019) Zenodo.

